# Spatial variability of lightning intensity over the Mediterranean sea correlates with seawater properties

**DOI:** 10.1038/s41598-023-33115-0

**Published:** 2023-04-10

**Authors:** Mustafa Asfur, Colin Price, Yoav Yair, Jacob Silverman

**Affiliations:** 1 Faculty of Marine Sciences, Ruppin Academic Center, Mikhmoret, Israel; 2 Porter School of the Environment and Earth Sciences, Tel Aviv University, Tel Aviv, Israel; 3 School of Sustainability, Reichman University, Herzliya, Israel; 4 National Institute of Oceanography, Israel Oceanographic and Limnological Research, Hubert Humphrey 1, Tel Shikmona 31080, Haifa, Israel

**Keywords:** Climate sciences, Ocean sciences, Planetary science

## Abstract

The divergence of total alkalinity (TA) from conservation with salinity (S) and relatively acidic conditions (pH) in surface seawater was suggested to explain the high prevalence of lightning superbolts in the Mediterranean sea, North sea and upwelling regions of the oceans. In this study we tested the combined effects of changes in S, TA and pH of Mediterranean sea surface water on the intensity of laboratory generated electrical sparks, which are considered to be analogous to cloud to sea-surface intensity of lightning discharges. The experimental results were used to develop a multivariate linear equation (MLE) of Lightning Flash Intensity (LFI) as a function of S, TA/S and pH. This relation was validated with wintertime (DJF) LFI measurements along a Mediterranean sea zonal profile during the period 2009–2020 compared to corresponding climate model outputs of S, TA and pH. Based on the resulting MLE, the combined effects of climate change, ocean acidification and the damming of the Nile, may have increased LFI in the Levantine Sea by 16 ± 14% until now relative to the pre-Aswan Dam period. Furthermore, assuming that salinization and acidification of the Levantine Sea will continue at current trends, the LFI is predicted to increase by 25 ± 13% by the year 2050.

## Introduction

Many previous studies proposed that lightning activity over the land and sea is primarily dependent on a number of intra-cloud processes, specifically, updraft, water droplet and ice particle size distribution and their frequency of collisions, which together cause the distribution and accumulation of electrical charge in the cloud^1,2^. Where, it has been assumed that the rate of charge distribution and accumulation, likely controls the frequency of lightning activity^3^, while the accumulated charge determines the intensity (peak current) of the electrical discharge^4,5^. Most of the previous studies focused on the geographical distribution of lightning activity, have considered only the frequency of lightning discharges. However, more recent studies have started to focus on the geographical variations in cloud to ground lightning intensity^6–9^. This was made possible, mainly with the development of observational systems capable of measuring lightning locations, times and peak currents or flash intensities on regional and global scales (World Wide Lightning Location Network—WWLLN^10^; Earth Networks Total Lightning Network—ENTLN^11^).

Recently, it was suggested that chemical properties of seawater, specifically, salinity, pH and total alkalinity (TA), may explain the observed prevalence of highly energetic electrical discharges, also known as lightning superbolts, over the oceans compared to land^6–8,12^. According to the Holzworth et al*.* global distribution of superbolts, it is apparent that the North Sea and the Mediterranean sea regions are hotspots of superbolt activity^12^. In fact, out of the 8171 superbolts included in the global dataset for the period 2010–2018, 1950 of them (nearly 25%) occurred over the Mediterranean sea (MS) region (over the water). Silverman et al*.*, suggested that the high density of superbolt activity over the MS could be explained by the high sea surface salinity and TA of this marginal sea^8^. In contrast, Pizzuti et al*.* ascribed the clustering of wintertime lightning superbolts over the sea surface in the northern English Channel during the period 2010–2020, to more favorable intracloud microphysical conditions in sea based compared to land-based storms, i.e., updraft, aerosol concentration and composition^9^.

**Figure 1:**
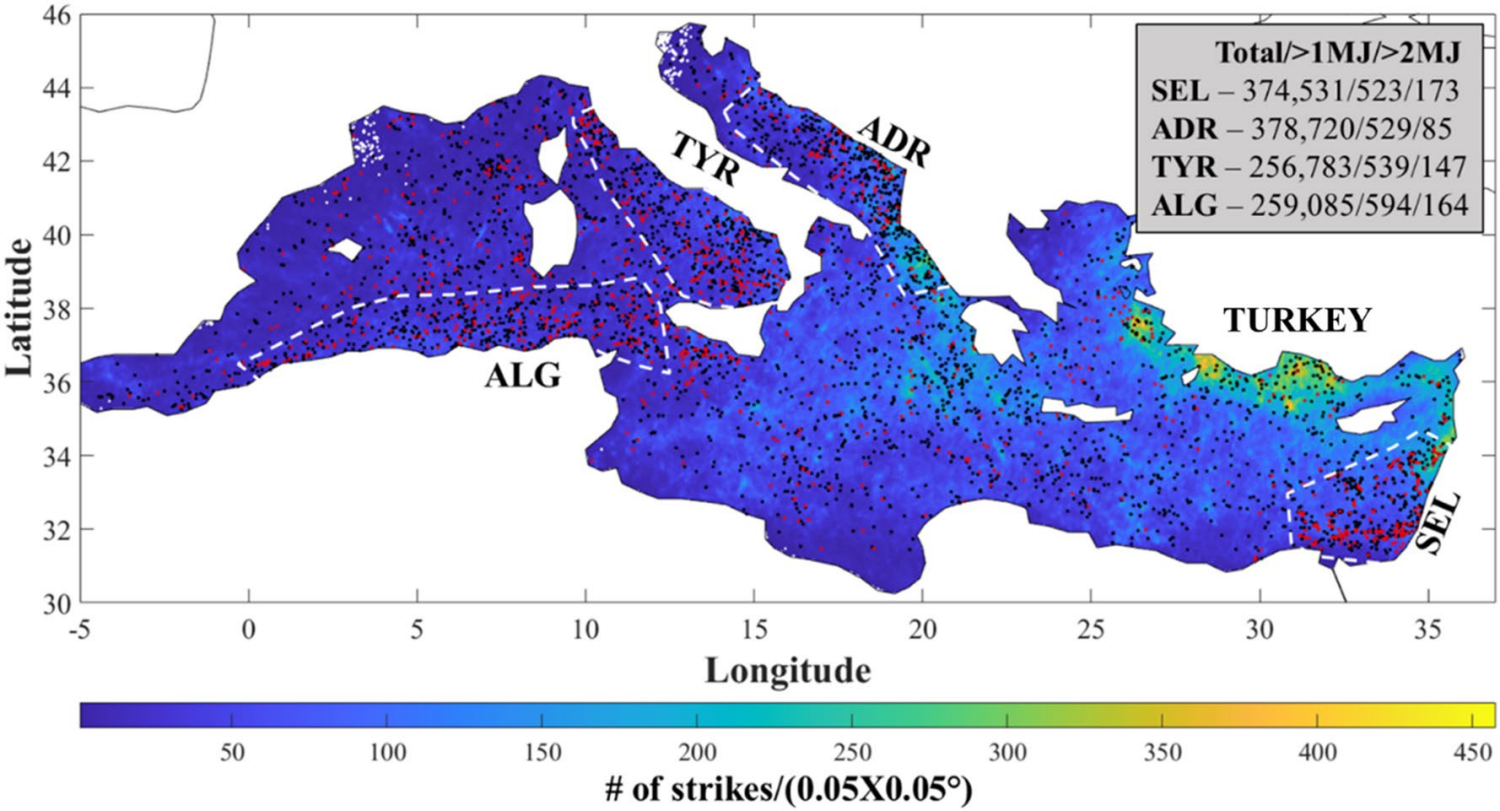
The spatial distribution of superbolts and cloud to ground lightning flash density over the Mediterranean sea during the winter months (DJF), observed by WWLLN over the period 2009–2021. The black and red dots indicate the locations of lightning flashes with energies > 10^6^ and 2 × 10^6^ Joules (superbolts), respectively. The dashed white lines indicate the regions with apparently higher frequencies of superbolt events in the Southeastern Levantine Sea (SEL), Southern Adriatic Sea (ADR), the Tyrrhenian Sea (TYR) and along the Mediterranean coast of Algeria (ALG). The numbers in the inset box indicate the total # of flashes/# of flashes with energies > 10^6^ Joules/# of flashes with energies > 2 × 10^6^ Joules, for each of the highlighted regions in the Mediterranean.

Mapping the geographical distribution of lightning superbolts detected by the WWLLN system during the winter months (DJF) of 2009–2021 over the MS, shows that there are four regions with clusters of superbolt activity (Fig. 1). These regions include the Southeastern Levantine Basin (SEL), the Adriatic and Tyrrhenian Seas (ADR and TYR, respectively) and along the Mediterranean coast of Algeria (ALG). It is interesting to note that in regions of relatively high flash density, such as along the Mediterranean coast of Turkey, the occurrence of superbolts is apparently lower.

Considering the observed distribution of superbolts over the MS in the context of the seawater properties, i.e., salinity, pH and TA, one would have expected a more coherent west to east increasing gradient in superbolt occurrence, in accordance with the observed gradients in salinity and TA^8^. Where, in the MS, the climatic wintertime distribution of sea surface salinity (SSS) shows an increase from ~ 37 PSU in the west to ~ 39 PSU in the east^13^. Correspondingly, the sea surface TA, which is conservative with salinity, also increases from a low value of ca. 2450 µmole/kg in the west to ca. 2650 µmole/kg in the east^14^. However, while TA does increase with salinity, the slope of increase in TA with SSS (ΔTA/ΔSSS) is much greater in the MS (~ 85 µmole/kg/PSU) than the slope in the open water of the Atlantic Ocean (~ 50 µmole/kg/PSU), which is the source of seawater for the MS^15,16^. Where, the higher TA to SSS slope in the MS has been attributed to terrestrial inputs of TA rich runoff (rivers and streams) and submarine groundwater discharge into the Mediterranean basin^14,17^. Similarly, it has been shown that CO_2_ enriched submarine groundwater discharge contributes to the acidification of coastal waters, in the MS and other similar coastal regions, such as Gulf of Mexico waters off the shore of Florida^18,19^. Thus, according to Asfur et al.^6^ and Silverman et al*.*^8^, which showed a positive correlation between laboratory generated electrical sparks salinity and TA, respectively, it would be expected that the intensity and occurrence of superbolts would increase from west to east in the MS. In Silverman et al*.*^8^, it was also suggested that the regions with relatively high densities of superbolts in the oceans correspond to regions that are strongly affected by terrigenous inputs of TA and Dissolved Inorganic Carbon (DIC = [CO2_(aq)_] + [HCO_3_^−1^] + [CO_3_^−2^]), and upwelling regions, where upwelled TA and DIC rich deep water, which are supersaturated with respect to atmospheric CO_2_, increases the TA of surface waters well above the expected SSS conservation value as well as significantly reducing their pH to the extent of being corrosive to CaCO_3_^20^. Thus, the clustering of superbolt activity in the different regions of the MS (Fig. 1) could also be related to local positive divergences of TA from SSS conservation and increased seawater acidification. In this study we investigated the combined effects of changes in pH, salinity (S) and TA of MS seawater on lightning intensity using the experimental setup of Asfur et al*.*^6,7^ and Silverman et al*.*^8^. Unlike, Asfur et al*.*^6,7^ and Silverman et al*.*^8^, the changes in salinity, total alkalinity and pH, tested in this study represent a more realistic scenario, specifically for the Mediterranean sea region, resulting from reduced freshwater input from the Nile, increased evaporation due to global warming and ocean acidification. Finally, unlike Asfur et al*.*^6,7^ and Silverman et al*.*^8^, the experimental relations observed in this study are tested on real world observations of lightning intensity from the WWLLN record (2009–2021) across the Mediterranean sea and show that they are significantly correlated with the spatial distribution of seawater properties (S, TA/S and pH) during the winter months (DJF).

## Materials and methods

During this study, electrical sparks were discharged from a cathode suspended in the air above a seawater sample with a submerged anode. The electrodes were connected to a power supply with a low output voltage of 2.8 V, which was connected to a high voltage step-up booster that produced an electrical potential of ~ 10^6^ V between the electrodes. The optical intensities of the electrical sparks that ionize the molecules in the air between the cathode and water surface were measured with a high frequency (100 kHz) spectrometer (100–900 nm). The spectrometer was connected to a fiberoptic sensor that relayed the light pulses from the optical emissions produced by the electrical discharges. The optical emission spectrum produced by the generated sparks are similar to those produced by natural lightning^8^ and therefore we use the parameter Lightning Flash Intensity (LFI) to describe the optical intensity of the electrical sparks in our experimental setup. See Asfur et al*.*^6,7^ and Silverman et al*.*^8^ for further details on the experimental setup.

Fresh MS seawater was collected one day before each experiment from the coastal waters off Michmoret, central Israel, with an initial salinity (S) of 39.095 ± 0.028 PSU, TA of 2621 ± 5 µmole/kg and pH_(T=25)_ of 8.063 ± 0.023 (n = 3) (Table SM1). Unlike Silverman et al*.*^8^, where TA was changed by addition of strong acid (HCl) or base (NaOH) with very little if any effect on S, in this study we changed the TA by dilution of the seawater samples with deionized water, which essentially simulate the dilution of seawater with meteoric fresh water. The fresh stock of filtered seawater collected before each experiment, was subsampled consecutively for each treatment into a 2 L graded Erlenmeyer Flask. After this we added to each flask varying volumes of distilled water as follows—25, 50, 70, 100, 125 and 150 mL, respectively. In addition to the dilution treatments, we amended the TA of the experimental solution after the initial seawater dilution with 50, 100 and 150 mL of deionized water by adding 160, 290, 420 µL NaOH (1 N), respectively. The volume of NaOH added was estimated in order to compensate for the loss of TA due to dilution back to its initial TA. This amendment was meant to simulate the addition of TA enriched freshwater from terrestrial runoff and submarine groundwater discharge (see above). The flasks were left in the lab overnight (24 h) while stirring gently with magnetic stirrers in order to accelerate the equilibration of CO_2_ in the experimental solution with ambient atmospheric CO_2_.

The following day, the solutions were subsampled into brown glass bottles through a 0.45 µm syringe GFF filter for later analysis in the lab of TA, DIC and density. The remaining solution was poured into our custom-made glass container, with a hole in the side for inserting the spectrometer fiber-optic sensor 1 cm above the waterline. Immediately afterwards, the container was placed in the lightning cabinet on a magnetic stirrer and the initial values of pH, temperature and conductivity of the experimental solution were measured with a handheld WTW multi-meter (MultiLine Multi 3630 IDS), while gently stirring the solution with a rotating magnetic bar. The pH was measured with a combination glass electrode and thermistore, which was calibrated at the beginning of every experiment day with WTW buffer solutions (pH 4.01 ± 0.02 and 7.00 ± 0.03). The specified precision of the electrode is ± 0.004 pH units. Conductivity was measured with a WTW-IDS digital conductivity cell TetraCon 925 electrode for universal applications (1 µS·cm^−1^–2000 mS·cm^−1^) with specified accuracy of ± 0.5%. After these measurements were taken, turning off the magnetic stirrer and closing the cabinet, we activated the low voltage power supply to generate electrical sparks for a period of 20 s and recording the spectral emission every 0.2 s.

After the initial measurement of the experimental solutions, we gently bubbled them for short periods (< 2 s) with CO_2_ gas (99.999%) to produce carbonic acid that caused a reduction in pH from its initial value of ca. pH ~ 8.2 and ~ 8.5 for NaOH treatments. After each bubbling period, the solution was gently stirred as described above to homogenize it, and pH was measured simultaneously until kinetic equilibrium was deemed to be attained when ΔpH/Δt <  + 0.001/5 s. Measurements of conductivity and temperature were also recorded for each bubbling step. The pH values that were tested by CO_2_ bubbling addition were in the range (6.5–8.5 at 25 °C). After each bubbling, the low voltage power supply was activated and the emission spectra of the generated sparks were repeatedly measured as described above. After the pH of the solution reached 6.5 the experiment was stopped and water samples were taken from the aquarium for later analysis in the lab of TA, DIC and density as described above.

Analyses of TA samples were done with a Methrom 785 Titrino Plus potentiometric titration system with ~ 0.05 N HCl, based on the analytical procedures and calculations described by Sass and Ben-Yaakov^21^. TA measurements were calibrated and standardized using seawater CRMs from Dickson’s lab^22^. DIC was measured by acidifying and stripping the CO_2_ gas from a subsample in a stream of high grade N_2_ gas (99.999%) using a custom-made system, AIRICA by Marianda gas distillation system. The distillation system transported the mixture to a LICOR 7000 IR absorbance sensor that integrated the partial pressure of CO_2_ over the entire measurement cycle. Replicate measurements were made (n = 3) for each sample and had an average precision of less than ± 2 µmol·kg^−1^. The system was adjusted for drift every 4 sample measurements with a Dickson CRM (Op. Cit.). Laboratory seawater density measurements were made with a 6-digit accuracy Anton Paar DMA-5000 densitometer and converted to salinity values with the measurement temperature using the equation of state for seawater from the 19th edition of standard methods^23^. The pH measurements were made at room temperature and were later adjusted to a constant temperature of 25 °C using the formula from Gieskes^24^.

## Results

All of the experimental treatment and results are presented in the supplementary online file (see link in the Acknowledgments section below) in Table SM1 and Fig. SM1. Our experimental results demonstrate a strong positive correlation between S and TA in the dilution treatments (MSW + DIW line in Fig. SM1a), while for the treatments where the TA of the experimental solution was adjusted with NaOH after dilution, the TA diverges increasingly with decreasing salinity and has a relatively constant value of ca. 2625 µmol·kg^−1^ (MSW + DIW + NaOH line in Fig. SM1a). In our experiments, we diluted the initial MS seawater to a minimum salinity and TA of ca. 36.4 PSU and 2631 ± 11 µmol·kg^−1^ (Fig. SM1a,b), respectively.

The LFI in our experiments was positively influenced by the TA and pH of the seawater (Fig. SM1b). However, despite the decrease in S due to dilution, the LFI was the highest for the experiments in which the TA of the solutions was adjusted by addition of NaOH to a value of 2631 ± 11 µmol·kg^−1^. These results suggest that the effect of varying TA overrides the influence of S on LFI as shown in Silverman et al*.*^8^. However, unlike the dependence of LFI on TA in Silverman et al*.*^8^, where ΔLFI/ΔTA =  − 0.03%/1 µmol·kg^−1^ in relative change units compared to the highest value, in this study the ΔLFI/ΔTA =  − 0.4%/1 µmol·kg^−1^. It should be noted that in Silverman et al*.*^8^, the TA was adjusted by additions of strong acid (HCl) or strong base (NaOH), which had very little effect on the conductivity or Salinity of the treated MS seawater. Therefore, it was concluded that LFI is less sensitive to changes in TA without corresponding changes in S due to dilution. The decoupling of the influence of S on LFI from TA is clearly demonstrated in Fig. SM1c. Where, despite the decrease in S due to dilution, adjustment of the TA back to its initial value by addition of NaOH has an increasingly stronger influence on LFI with decreasing S.

**Figure 2:**
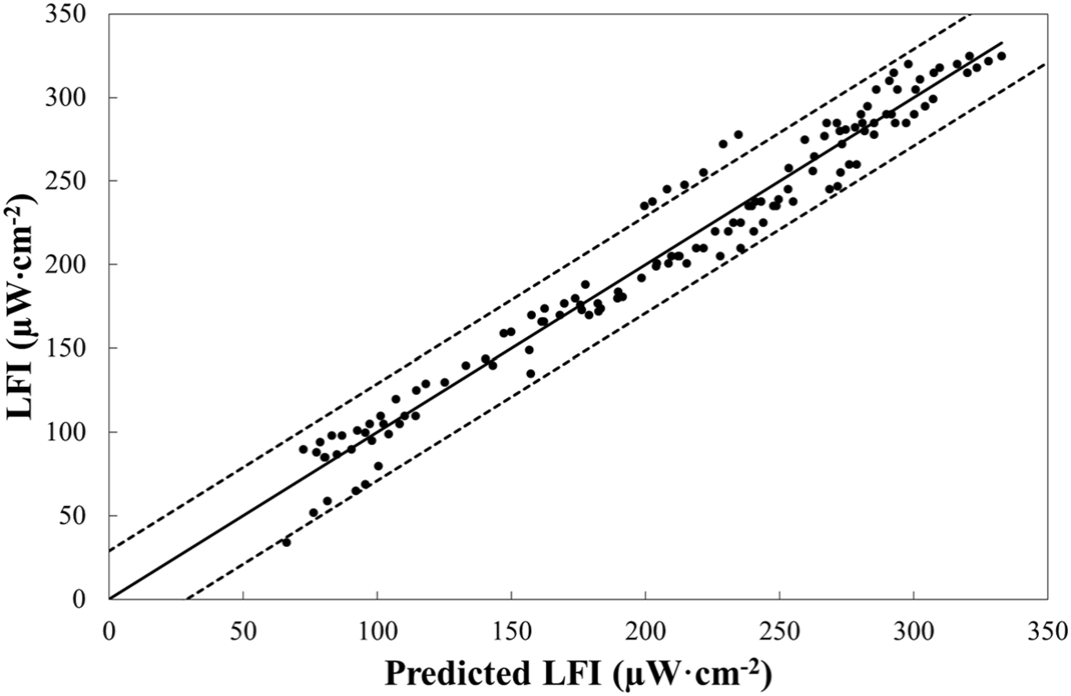
Comparison of experimental LFI and predicted LFI values using a MLE as a function of the measured S, pH and TA/S, where the Predicted LFI =  − 22.4 (± 2.3)·pH + 57.8(± 1.4)·S + 37.7 (± 0.7)·(TA/S) − 4367 (± 85.6) (n = 132; R^2^ = 0.96; p < 0.0001). In the equation for predicted LFI, the numbers in parentheses are the standard errors of the parameter coefficients and the intercept. The regression line is represented by the continuous black line and the dashed black lines represents the upper and lower 95% confidence interval range.

In real world terms, TA and S are strongly and positively correlated, however the ratio of TA to S (TA/S) of surface water in specific regions of the oceans may vary considerably above or below the Salinity Dilution Line (SDL) due to addition or removal of TA^17,25^ from external sources or within the water column. Thus, the results so far suggest that besides the effects of S and pH on LFI, the ratio TA/S will contribute to the LFI as well. Where, TA/S appears to contribute positively to LFI according to our experimental results. Thus, the Multivariate Linear Equation (MLE) of LFI as a function of the experimental conditions of S, pH and TA/S, yields a highly significant fit (p << 0.0001) as seen in the comparison between the measured LFI and the calculated LFI (Fig. 2). Where, the leverage residuals for LFI indicate a highly significant positive relation with S as expected^6^, a highly significant negative relation of LFI with pH as expected^7^ and a highly significant positive relation with TA/S (Table SM1). Note that these relations are demonstrated only for MS seawater.

## Discussion and conclusions

In 2016, the World Meteorological Organization declared that lightning is an essential climate variable^26^. To date, with the exception of Asfur et al*.*^7^, global change studies have only considered the effect of warming on lightning flash frequency and the global distribution of lightning activity^27–30^. Price and Rind^26^ and Williams^29^ based their predictions of increased lightning frequency on theoretical considerations, while Reeve and Toumi^27^ and Romps et al*.*^30^ based their predictions on observed relations between spatial variations of flash frequency, air temperature and precipitation derived from satellite measurements over an annual cycle. Furthermore, none of these studies considered the effects of warming on lightning flash intensity. Therefore, it is still difficult to determine the effects of global climate change on the intensity of lightning flashes with any reasonable degree of certainty from observations.

**Figure 3:**
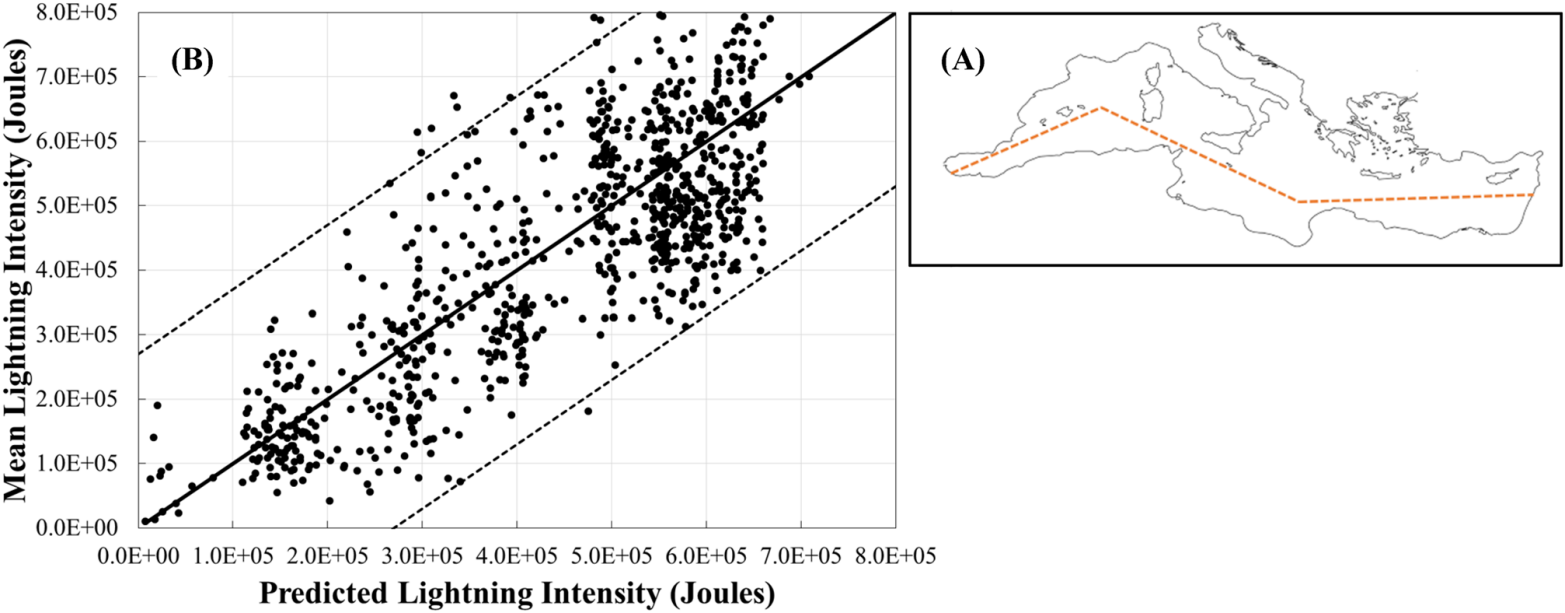
Comparison of mean lightning intensities along the Mediterranean sea zonal profile (**A**), determined from the WWLLN measurements during the winter months (December, January and February) over the period 2009–2021, and predicted lightning intensities using a MLE as a function of the surface water S, pH and TA/S from the Copernicus dataset for the corresponding months over the same period (**B**). Where, Predicted Lightning Intensity =  − 7,135,883 (± 771,242)·pH + 37,662 (± 9328)·S + 437,509 (± 22,667)·(TA/S) + 27,233,671 (± 5,371,319) (n = 943; R^2^ = 0.61; p < 0.0001). The numbers in parentheses in the equation, are the standard errors of the parameter coefficients and the intercept. The regression line is represented by the continuous black line and the dashed black lines represents the upper and lower 95% confidence interval range.

The MLE, developed to predict the LFIs observed in our experimental setup from the salinity (S), pH and the TA/S ratio of seawater (Fig. 2), was used to predict the LFI of lightning intensities along a zonal profile crossing the Mediterranean from west to east (Fig. 3A). Where, monthly (DJF) average values of surface water S, pH and TA in corresponding grid points along the profile were extracted from the Copernicus web site (https://marine.copernicus.eu/) for the period 2009–2020. Similarly, monthly (DJF) averages of lightning intensities that were recorded by the WWLLN system over the period along the cross Mediterranean profile were calculated. In Fig. 3B, we present the significant and positive relations between the calculated monthly mean values and the calculated lightning intensities in corresponding grid cells along Mediterranean Sea zonal profile. Interestingly, the strongest effect on the MLE lightning was due to the TA/S ratio, followed by pH and S, which had the least effect. Additionally, the TA/S and S had a positive effect on the predicted lightning intensity, while the pH had a negative effect. These outcomes are very similar to the experimental results of this study (Fig. 2). Thus, the relatively strong correlation and the effect directions support the hypothesis that lightning intensity over seawater may be influenced also by the seawater chemical properties considered in this study.

In the context of this study, it is interesting to note that the regions of high lightning superbolt activity in the Mediterranean Sea (Fig. 1) and the English Channel^9^, are also regions that have positive TA anomalies with respect to their corresponding salinities in the surface seawater^14,17,31–33^. Where, the excess of TA in the English Channel and relatively low winter time pH could be associated with TA and dissolved inorganic carbon rich groundwater discharge and terrestrial surface runoff inputs from the surrounding land masses and the relatively small volume of this water body. Additionally, alkalinity and dissolved inorganic carbon released from the anoxic bottom sediments, due to sulfate reduction and dissolution of carbonates, in the relatively shallow waters of the North Sea and northern English Channel^34^ may also contribute significantly to the anomalous carbonate system conditions relative to salinity and enhance lightning flash intensity as well. Similarly, in the southern Adriatic Sea, such anomalous carbonate system conditions also occur during the winter months for the similar reasons^35^, while in the Tyrrhenian Sea, the anomalous carbonate system levels are likely related to the volcanic and hydrothermal activity in the region^36^.

The mean pH, S and TA/S ratio in the Tyrrhenian Sea according to the measurements of Mishra et al*.*^36^ near hydrothermal seeps and their reference sites, which were not significantly different, are 7.9 ± 0.3, 36.2 ± 0.5 PSU and 72 ± 3 µmol·kg^−1^·PSU^−1^, respectively. Similarly, the mean values for pH, S and TA/S ratio during the winter season across the southern Adriatic according to the measurements reported in Cantoni et al*.*^35^ are 38.2 ± 0.5 PSU, 8.1, 69 ± 2 µmol·kg^−1^·PSU^−1^, respectively, with a maximum value of 73 µmol·kg^−1^·PSU^−1^. The predicted LFIs using the MLE based on the experimental results (Fig. 2) are 263, 261 and 234 µW·cm^−2^ for TYR, ADR and SEL, respectively. Where, the data used to calculate the predicted LFI for SEL was taken from Kolker et al*.*^17^. Unfortunately, wintertime measurements of the surface water carbonate system along the Algerian coast of Africa in the MS (ALG in Fig. 1), are not readily available in the scientific literature and therefore we cannot currently validate our MLE for LFI in this region.

Present and future sea surface warming of Mediterranean seawater could increase convection and charge accumulation in thunder clouds, potentially resulting in increased lightning flash frequency^37^. Furthermore, considering the unique influence of climate change and other anthropogenic influences on the Mediterranean Sea water, salt, total alkalinity and dissolved inorganic carbon budgets, our experimental results and their confirmation suggest that lightning intensity may have already increased and may continue to do so with additional future warming and ocean acidification.

According to a modeling study^38^, the significant reduction of freshwater input from the Nile into the Mediterranean following the erection of the Aswan High Dam (AHD) caused the salinity in the upper 0–20 m in the eastern Mediterranean to increase from ~ 38.95 pre damming to ~ 39.1 PSU less than 10 years post damming. In addition, seasonal measurements in the open waters of the eastern Mediterranean during the period 1979–2014 have shown that the annual mean salinity of the LSW increased from ~ 39.2 to 39.5 PSU^39^. Thus, since the damming of the Nile in 1964, the overall increase in the salinity of the eastern Mediterranean was at least 0.4 PSU. Bialik and Sisma-Ventura^40^ estimated based on available measurements of salinity in the surface water of the eastern Mediterranean since 1947 until 2010 that the wintertime salinity increased from 38.74 to 38.87 pre-AHD (1947–1964) to 39.08–39.40 in the post-AHD period (1980–2010). Thus, according to Bialik and Sisma-Ventura^40^, the resulting increase in TA during the wintertime over the same period was 2594–2596 to 2616–2635 µmol·kg^−1^ (ΔTA = 41 µmol·kg^−1^). Furthermore, based on a reconstructed record of DIC in the surface water of the eastern Mediterranean from measurements of δ^13^C in Vermetid reef cores and estimated TAs (from salinity), they calculated the change in pH over the same period, which yielded an overall decrease in the pH of LSW from ~ 8.1 to ~ 8 at an average rate of − 0.022 ± 0.002 decade^−1^. This rate is slightly higher than the rate of acidification in oceanic surface waters due to the anthropogenic increase in atmospheric CO_2_^41^.

Using the experimental MLE (Fig. 2), the overall effect of climate change, ocean acidification and the AHD on LFI in the eastern Mediterranean sea until now is an increase of 16 ± 14% relative to the pre-1960 LFI. Assuming that salinization and acidification of LSW will continue at current trends, the LFI will increase by 25 ± 13% in 2050 relative to the pre-1960s. Alternatively, according to the predicted changes in sea surface salinity of the Mediterranean Sea (ca. + 0.9 PSU since AHD), based on the ensemble of climate change scenario simulations according to Adloff et al*.*^42^ and the current ocean acidification trend, LFI will increase by ca. 35 ± 12% by 2100 relative to pre-1960 LFI. However, it should be noted that a more recent coupled ocean–atmosphere ensemble modeling study suggests that increased flow of relatively fresher northern Atlantic Ocean water through the Gibraltar Strait into the Mediterranean Sea will substantially subdue current salinization trends by the end of the twenty-first century^43^. Finally, a further complication that may affect these predictions is the potential increase in TA resulting from climate mediated increases in karst activity around the Mediterranean land boundaries. Where, the increased dissolution of CaCO_3_ in karst systems, which discharge through runoff and submarine groundwater discharge into the Mediterranean, due to warming and increasing atmospheric CO_2_^44^. This feedback mechanism may increase the TA/S as well as acidification in the Mediterranean Sea resulting in an additional increase in lightning intensity as well.

In conclusion, the experimental results of this study show for the first time that the combined effect of changes in S, TA and pH of Mediterranean seawater mediated by dilution, strong base addition and CO_2_ bubbling, have a strong influence on the intensity of electrical sparks (LFI) discharged into experimental seawater solutions. Furthermore, the relation between LFI and S was decoupled from its positive dependence on S, when the diluted seawater’s TA was amended by addition of the strong base, demonstrating that excess TA with respect to it conservation with S (TA/S) has a strong positive influence on LFI. The resulting experimental relation between S, TA/S, pH and LFI in our experimental setup was tested and significantly verified with actual measurements of lightning intensity over the Mediterranean sea and corresponding seawater properties. Thus, changes in these seawater properties resulting from climate change and other anthropogenic impacts on the salt, carbon and alkalinity budgets of the Mediterranean Sea could and may already have caused lightning intensity over the sea surface to increase. Finally, considering that the Mediterranean Sea is a hotspot for lightning superbolt activity and climate change, it is important to monitor, not only trends in lightning flash frequency, but also lightning intensity as an essential climate variable.

## Supplementary Information

Supplementary Information.

## Data Availability

All experimental data presented in this report are available in the supplementary online material and the Mendeley Data repository at https://doi.org/10.17632/bndsdtcz3r. World Wide Lightning Location Network (WWLLN) data used in this study was attained upon request from Prof. Holzworth through the website—http://wwlln.net/.
